# Investigation of the genetic aetiology of Lewy body diseases with and without dementia

**DOI:** 10.1093/braincomms/fcae190

**Published:** 2024-05-31

**Authors:** Lesley Yue Wu, Raquel Real, Alejandro Martinez-Carrasco, Ruth Chia, Michael A Lawton, Maryam Shoai, Catherine Bresner, Cornelis Blauwendraat, Andrew B Singleton, Mina Ryten, International Lewy Body Dementia Genomics Consortium, Yevgeniya Abramzon, Sarah Ahmed, Camille Alba, Marilyn S Albert, Dagmar Bacikova, Matthew J Barrett, Thomas G Beach, David A Bennett, Lilah M Besser, Eileen H Bigio, Bradley F Boeve, Ryan C Bohannan, Chad A Caraway, Jose-Alberto Palma, Ruth Chia, Clifton L Dalgard, Dennis Dickson, Jinhui Ding, Kelley Faber, Tanis Ferman, Luigi Ferrucci, Margaret E Flanagan, Tatiana M Foroud, Bernardino Ghetti, J Raphael Gibbs, Alison Goate, David Goldstein, Neill R Graff-Radford, Heng-Chen Hu, Daniel Hupalo, Scott M Kaiser, Horacio Kaufmann, Ronald C Kim, Gregory Klein, Walter Kukull, Amanda Kuzma, James Leverenz, Grisel Lopez, Qinwen Mao, Elisa Martinez-McGrath, Eliezer Masliah, Ed Monuki, Kathy L Newell, Lucy Norcliffe-Kaufmann, Matthew Perkins, Olga Pletnikova, Alan E Renton, Susan M Resnick, Owen A Ross, Marya S Sabir, Clemens R Scherzer, Sonja W Scholz, Geidy Serrano, Vikram Shakkotai, Ellen Sidransky, Andrew B Singleton, Toshiko Tanaka, Nahid Tayebi, Bryan J Traynor, Juan C Troncoso, Coralie Viollet, Ronald L Walton, Randy Woltjer, Zbigniew K Wszolek, Sandra E Black, Ziv Gan-Or, Julia Keith, Mario Masellis, Ekaterina Rogaeva, Dag Aarsland, Safa Al-Sarraj, Johannes Attems, Raffaele Ferrari, Steve Gentleman, John A Hardy, Angela K Hodges, Seth Love, Ian McKeith, Christopher M Morris, Huw R Morris, Laura Palmer, Stuart Pickering-Brown, Regina H Reynolds, Mina Ryten, Alan J Thomas, Bension S Tilley, Claire Troakes, Francesca Brett, Alexis Brice, Charles Duyckaerts, Suzanne Lesage, Maura Brunetti, Andrea Calvo, Antonio Canosa, Adriano Chiò, Gianluca Floris, Giancarlo Logroscino, Chiara Zecca, Jordi Clarimon, Monica Diez-Fairen, Juan Fortea, Isabel González-Aramburu, Jon Infante, Carmen Lage, Alberto Lleó, Pau Pastor, Laura Porcel-Molina, Eloy Rodríguez-Rodríguez, Pascual Sanchez-Juan, Rejko Krüger, Patrick May, Georgia Xiromerisiou, Sonja W Scholz, Bryan J Traynor, Nigel M Williams, Michele T M Hu, Yoav Ben-Shlomo, Donald G Grosset, John Hardy, Huw R Morris

**Affiliations:** Department of Clinical and Movement Neurosciences, UCL Queen Square Institute of Neurology, University College London, London WC1N 3BG, UK; UCL Movement Disorders Centre, University College London, London WC1N 3BG, UK; Aligning Science Across Parkinson’s (ASAP) Collaborative Research Network, Chevy Chase, MD 20815, USA; Department of Clinical and Movement Neurosciences, UCL Queen Square Institute of Neurology, University College London, London WC1N 3BG, UK; UCL Movement Disorders Centre, University College London, London WC1N 3BG, UK; Aligning Science Across Parkinson’s (ASAP) Collaborative Research Network, Chevy Chase, MD 20815, USA; Department of Clinical and Movement Neurosciences, UCL Queen Square Institute of Neurology, University College London, London WC1N 3BG, UK; UCL Movement Disorders Centre, University College London, London WC1N 3BG, UK; Aligning Science Across Parkinson’s (ASAP) Collaborative Research Network, Chevy Chase, MD 20815, USA; Neuromuscular Diseases Research Section, Laboratory of Neurogenetics, National Institute on Aging, Bethesda, MD 20814, USA; Population Health Sciences, Bristol Medical School, University of Bristol, Bristol BS8 2PS, UK; Aligning Science Across Parkinson’s (ASAP) Collaborative Research Network, Chevy Chase, MD 20815, USA; Department of Neurodegenerative Diseases, UCL Queen Square Institute of Neurology, University College London, London, WC1N 3BG, UK; UK Dementia Research Institute, University College London, London WC1E 6BT, UK; Institute of Psychological Medicine and Clinical Neurosciences, MRC Centre for Neuropsychiatric Genetics and Genomics, Cardiff University, Cardiff CF24 4HQ, UK; Integrative Neurogenomics Unit, National Institute on Aging, Bethesda, MD 20814, USA; Center for Alzheimer’s and Related Dementias, National Institute on Aging, Bethesda, MD 20892, USA; Center for Alzheimer’s and Related Dementias, National Institute on Aging, Bethesda, MD 20892, USA; Aligning Science Across Parkinson’s (ASAP) Collaborative Research Network, Chevy Chase, MD 20815, USA; Genetics and Genomic Medicine, UCL Great Ormond Street Institute of Child Health, University College London, London WC1N 1EH, UK; Genetics and Genomic Medicine, NIHR Great Ormond Street Hospital Biomedical Research Centre, University College London, London WC1N 1EH, UK; UK Dementia Research Institute at The University of Cambridge, Cambridge, UK; Department of Clinical Neurosciences, School of Clinical Medicine, The University of Cambridge, Cambridge, UK; Neurodegenerative Diseases Research Section, National Institute of Neurological Disorders and Stroke, Bethesda, MD 20892, USA; Department of Neurology, Johns Hopkins University Medical Center, Baltimore, MD 21287, USA; Neuromuscular Diseases Research Section, Laboratory of Neurogenetics, National Institute on Aging, Bethesda, MD 20814, USA; Department of Neurology, Johns Hopkins University Medical Center, Baltimore, MD 21287, USA; Reta Lila Weston Institute, UCL Queen Square Institute of Neurology, London WC1N 1PJ, UK; Institute of Psychological Medicine and Clinical Neurosciences, MRC Centre for Neuropsychiatric Genetics and Genomics, Cardiff University, Cardiff CF24 4HQ, UK; Nuffield Department of Clinical Neurosciences, Division of Clinical Neurology, University of Oxford, Oxford OX3 9DU, UK; Oxford Parkinson’s Disease Centre, University of Oxford, Oxford OX1 3QU, UK; Population Health Sciences, Bristol Medical School, University of Bristol, Bristol BS8 2PS, UK; School of Neuroscience and Psychology, University of Glasgow, Glasgow G12 8QQ, UK; Aligning Science Across Parkinson’s (ASAP) Collaborative Research Network, Chevy Chase, MD 20815, USA; Department of Neurodegenerative Diseases, UCL Queen Square Institute of Neurology, University College London, London, WC1N 3BG, UK; UK Dementia Research Institute, University College London, London WC1E 6BT, UK; Reta Lila Weston Institute, UCL Queen Square Institute of Neurology, London WC1N 1PJ, UK; National Institute for Health Research (NIHR) University College London Hospitals Biomedical Research Centre, London W1T 7DN, UK; Institute for Advanced Study, The Hong Kong University of Science and Technology, Hong Kong SAR, China; Department of Clinical and Movement Neurosciences, UCL Queen Square Institute of Neurology, University College London, London WC1N 3BG, UK; UCL Movement Disorders Centre, University College London, London WC1N 3BG, UK; Aligning Science Across Parkinson’s (ASAP) Collaborative Research Network, Chevy Chase, MD 20815, USA

**Keywords:** Lewy body diseases, dementia, genome-wide association studies, *APOE*

## Abstract

Up to 80% of Parkinson's disease patients develop dementia, but time to dementia varies widely from motor symptom onset. Dementia with Lewy bodies presents with clinical features similar to Parkinson’s disease dementia, but cognitive impairment precedes or coincides with motor onset. It remains controversial whether dementia with Lewy bodies and Parkinson's disease dementia are distinct conditions or represent part of a disease spectrum. The biological mechanisms underlying disease heterogeneity, in particular the development of dementia, remain poorly understood, but will likely be the key to understanding disease pathways and, ultimately, therapy development. Previous genome-wide association studies in Parkinson's disease and dementia with Lewy bodies/Parkinson's disease dementia have identified risk loci differentiating patients from controls. We collated data for 7804 patients of European ancestry from Tracking Parkinson’s, The Oxford Discovery Cohort, and Accelerating Medicine Partnership—Parkinson's Disease Initiative. We conducted a discrete phenotype genome-wide association study comparing Lewy body diseases with and without dementia to decode disease heterogeneity by investigating the genetic drivers of dementia in Lewy body diseases. We found that risk allele rs429358 tagging *APOEe4* increases the odds of developing dementia, and that rs7668531 near the *MMRN1* and *SNCA-AS1* genes and an intronic variant rs17442721 tagging *LRRK2* G2019S on chromosome 12 are protective against dementia. These results should be validated in autopsy-confirmed cases in future studies.

## Teaser

Lewy body diseases are heterogeneous and could present with or without dementia. Wu *et al*. conducted a case–case genome-wide association study in 7804 patients and identified variant tagging *APOE e4* to increase odds of developing dementia and variants near *SNCA-AS1* and tagging *LRRK2* to be protective.

## Graphical Abstract

**Graphical Abstract:**
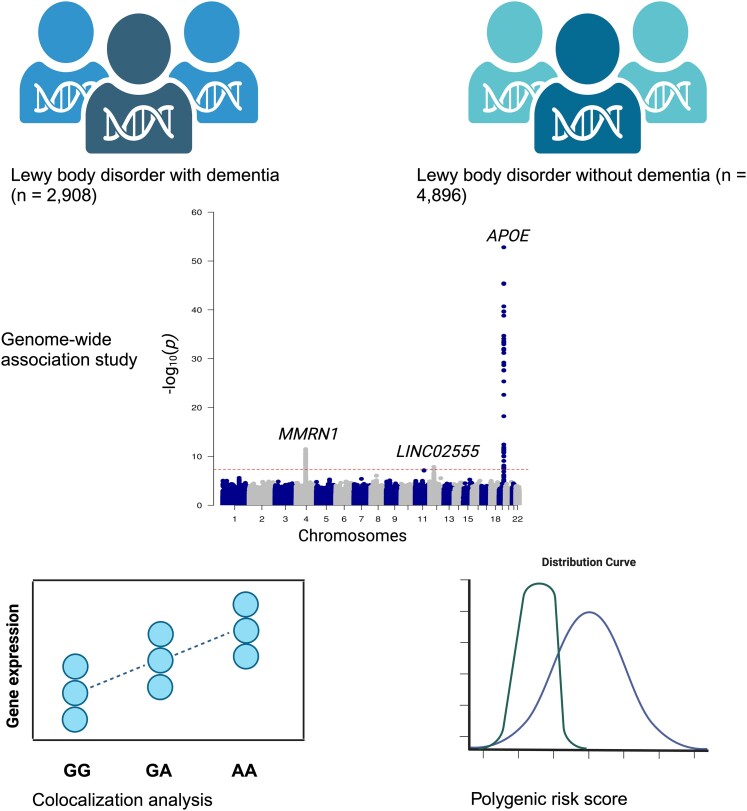

## Introduction

Parkinson's disease, Parkinson’s disease dementia and dementia with Lewy bodies, which we describe here jointly as Lewy body diseases, are characterized pathologically by alpha-synuclein aggregates forming Lewy bodies and Lewy neurites.^1^ Parkinson’s disease is a common degenerative movement disorder presenting with tremor, rigidity and bradykinesia. Non-motor features, including cognitive impairment and dementia, develop with disease progression in Parkinson’s disease. Approximately 24% of Parkinson’s disease patients have mild cognitive impairment at the time of diagnosis,^2^ and up to 80% of Parkinson’s disease patients eventually progress to dementia (Parkinson’s disease dementia),^3^ which is associated with worse functioning, poorer quality of life, care home admission and significant morbidity.^4^ However, the time to dementia from motor symptom onset varies widely between patients. Dementia with Lewy bodies is a synucleinopathy presenting with symptoms similar to Parkinson’s disease dementia, including dementia, cognitive fluctuations, visual hallucinations and REM sleep behaviour disorder in conjunction with existing or latent parkinsonism.^5^ Clinically, Parkinson’s disease dementia and dementia with Lewy bodies are distinguished by the ‘1-year rule’, where Parkinson’s disease dementia is diagnosed when dementia develops in the context of well-established Parkinson’s disease more than 1 year after motor symptom onset, while a diagnosis of dementia with Lewy bodies is given when cognitive impairment precedes or coincides with motor impairment. Parkinson’s disease dementia is distinguished from dementia with Lewy bodies by the temporal sequence of symptoms.

Neuropathologically, Parkinson’s disease usually differs from Parkinson’s disease dementia/dementia with Lewy bodies in the extent of Lewy body pathology in the brain, as inclusions are limited to the limbic system or brainstem in Parkinson’s disease without dementia. However, the pathological delineation of Parkinson’s disease dementia from dementia with Lewy bodies is extremely difficult. Both are characterized by Lewy bodies in cortical areas and a high frequency of Alzheimer’s disease co-pathology. Indeed, about 50% of Parkinson’s disease dementia patients have beta-amyloid plaques and neurofibrillary tangles at postmortem, which may be a better predictor of dementia than the extent of cortical alpha-synuclein pathology.^6^ The majority of dementia with Lewy bodies brains also fulfil criteria for a secondary diagnosis of Alzheimer’s disease.^7^ While a recent pathological study examining 110 Parkinson’s disease dementia and 78 dementia with Lewy bodies postmortem brains showed more severe synuclein cortical load, Alzheimer’s disease-related pathological changes and cerebral amyloid angiopathy in the dementia with Lewy bodies brains,^8^ it is generally agreed that there are no clear hallmark features distinguishing the two diseases.^9^ The separation of Parkinson’s disease dementia and dementia with Lewy bodies as discrete clinical and pathological entities is controversial.

Lewy body diseases are primarily sporadic. Case–control genome-wide association studies (GWAS) in the past decade have identified 90 common variant risk loci associated with Parkinson’s disease^10^ and 5 risk loci associated with dementia with Lewy bodies.^11^ Variation in several genes, including *GBA1*, *TMEM175* and *SNCA*, confers risk for both diseases, suggesting overlapping pathogenesis and underlying biological dysfunction. Strikingly, *TMEM175* and *SNCA* also modulate age at onset in Parkinson’s disease.^12^ On the other hand, there are distinct loci for dementia with Lewy bodies compared with Parkinson’s disease encompassing different genes (e.g. *APOE* and *BIN1* for dementia with Lewy bodies), and, in some cases, distinct association signals at the same locus. In a study using targeted high-throughput sequencing, two distinct regions of the *SNCA* gene at the 3′ and 5′ ends were found to be differentially associated with Parkinson’s disease and dementia with Lewy bodies risk, respectively.^13^ While the consequences of these distinct signals remain to be clarified, it has been hypothesized that these distinct association signals could relate to the control of gene expression in different brain regions, leading to different phenotypes.^14^ Genome-wide survival analysis of Parkinson’s disease identified *RIMS2*^15^ and *LRP1B*^16^ as common risk loci for progression from Parkinson’s disease to Parkinson’s disease dementia; however, they do not seem to be relevant to dementia with Lewy bodies.

Heterozygous mutations in *GBA1* are among the strongest genetic risk factors for Parkinson’s disease and dementia with Lewy bodies.^17,18^  *GBA1* encodes glucocerebrosidase, a lysosomal enzyme involved in the metabolism of glycosphingolipid. A meta-analysis of Parkinson’s disease patients showed that *GBA1* mutations are associated with a 2.4-fold increase in the incidence of cognitive impairment.^19^ Moreover, mutation carriers tend to have earlier disease onset^12^ and shorter survival.^20^ In a large multicentre study of *GBA1* mutation carriers, *GBA1* was also found to be strongly associated with Parkinson’s disease dementia as well as dementia with Lewy bodies, providing evidence that *GBA1* mutations lead to impaired cognition in synucleinopathies.^21^ However, as is the case for *SNCA*, the specific variants associated with Parkinson’s disease and dementia with Lewy bodies differ.^22^ Although the role of these *GBA1* variants in pathogenesis remains unclear, studies in postmortem tissue showed that reduced lysosomal GCase is associated with alpha-synuclein aggregation, inflammation and cellular damage,^22^ suggesting an important role for GCase in the propagation of the alpha-synuclein pathology. This could explain the spread of Lewy bodies to limbic and neocortical areas of Parkinson’s disease patients with *GBA1* mutations.

The apolipoprotein E (*APOE*) ε4 allele, a well-known risk locus for Alzheimer’s disease, has also been identified as a strong genetic risk factor for developing Parkinson’s disease dementia/dementia with Lewy bodies.^11^  *APOE4* promotes amyloid-beta oligomerization and its pathological accumulation.^23^ The role of *APOE4* in dementia with Lewy bodies pathogenesis is still unclear. It has been suggested that *APOE4* might be a driver of amyloid-beta deposition, which presents as a co-pathology in the majority of dementia with Lewy bodies brains.^7^ However, there is some evidence showing that *APOE* may contribute to cognitive decline independently of amyloid. In autopsy studies, *APOE4* was associated with dementia and diffuse LB pathology in ‘pure’ dementia with Lewy bodies patients (i.e. with absent or low levels of amyloid) as well as Parkinson’s disease dementia.^24,25^ Mouse models of synucleinopathy have also demonstrated that *APOE4* exacerbated alpha-synuclein pathology in the absence of amyloid.^26,27^

Parkinson’s disease, Parkinson’s disease dementia and dementia with Lewy bodies share common risk genes. However, specific risk loci within these genes may vary across diseases, potentially leading to different phenotypes, which ultimately relate to the involvement of different cell types. Previous GWAS for Parkinson’s disease and dementia with Lewy bodies have compared Parkinson’s disease and dementia with Lewy bodies cases with controls. Here, in a study of almost 8000 cases, we aim to define the genetic determinants of dementia in Lewy body diseases by taking a different approach. We have taken a disease classification agnostic approach by comparing all Lewy body diseases with dementia (LBD-D), including both Parkinson’s disease dementia and dementia with Lewy bodies, to Parkinson's disease cases without dementia (LBD-ND). This ‘case–case’ approach should help identify specific variants that are associated with more extensive LB and Alzheimer’s disease pathology that contribute to cognitive impairment, rather than variants that are related to the initiation of the LB pathology as compared with unaffected controls.

## Materials and methods

### Cohort description and study design

We analysed three large independent cohorts: Tracking Parkinson's (TPD, www.parkinsons.org.uk/),^28^ Oxford Parkinson's Disease Centre Discovery (OPDC, www.dpag.ox.ac.uk/opdc/)^29^ and Accelerating Medicine Partnership—Parkinson's Disease Initiative (AMP-PD v2.5, https://www.amp-pd.org/) (Table 1, Supplementary Table 1). The AMP-PD data set is enriched for patients with *LRRK2* p.G2019S. Participants were included in the present study based on their most recent clinical diagnosis or final pathological diagnosis of Parkinson’s disease, Parkinson’s disease dementia or dementia with Lewy bodies. A status of ‘case’ for LBD-D was defined if the patient had a clinical diagnosis of dementia with Lewy bodies^5^ or met the Movement Disorder Society task force Parkinson’s disease dementia diagnostic criteria.^30^ In detail, for Parkinson’s disease dementia, the criteria included (i) scoring below the threshold for dementia on the Montreal Cognitive Assessment (MoCA score < 21/30); (ii) having cognitive deficits that are severe enough to interfere with activities of daily living (MDS-Unified Parkinson's disease Rating Scale (UPDRS) part I 1.1 ≥ 2 score) and (iii) and the absence of severe depression defined using the MDS-UPDRS (MDS-UPDRS part I 1.3 < 4). LBD-ND was given a status of ‘control’. These patients did not have dementia based on the available clinical data. Patients with a change of diagnosis to a non-Lewy body disorder during the follow-up period were removed from analyses. AMP-PD is a unified cohort consisting of eight longitudinal studies with similar sample collection protocols. All studies were approved by local and multicentre ethics committees and are in compliance with the Declaration of Helsinki. Appropriate data use agreements were approved.

**Table 1:** Cohort demographics

Cohort	TPD		OPDC		AMP-PD		Total	
	LBD-D	LBD-ND	LBD-D	LBD-ND	LBD-D	LBD-ND	LBD-D	LBD-ND
*N*	159	1377	93	737	2656	2782	2908	4896
*N* male (%)	128 (81)	863 (63)	72 (77)	460 (62)	1694 (64)	1695 (61)	1894 (65)^a^	3017 (61)
Age diagnosis, years	69.0 (7.9)	64.3 (9.9)	68.2 (9.0)	64.2 (9.6)	76.5 (8.8)	60.4 (10.5)	75.8 (9.1)^b^	62.1 (10.4)
Array/sequencing	Illumina HumanCore Exome array		Illumina HumanCore Exome-12 v1.1 or Illumina InfiniumCoreExome-24 v1.1		Illumina HiSeq X Ten			

Means (SD) are shown unless otherwise indicated. Data shown are only in individuals who had both clinical and genetic data after quality control filters have been applied within each cohort.

^a^There are significantly more males in the LBD-ND group (*P* = 2.31e^−16^).

^b^LBD-D is significantly older than LBD-BD (*P* = 2e^−16^).

TPD, Tracking Parkinson's; OPDC, Oxford Parkinson's Disease Centre Discovery, Accelerating Medicine Partnership—Parkinson's Disease Initiative; LBD-D: Lewy body disease with dementia; and LBD-ND: Lewy body disease without dementia.

### Genotyping and quality control

DNA was extracted from whole blood or brain tissue as detailed in the protocols of each study. TPD used the Illumina HumanCoreExome array for genotyping. OPDC generated genotype data using the Illumina HumanCoreExome-12 v1.1 and Illumina Infinium HumanCoreExome-24 v1.1 SNP arrays. Whole genome sequencing for AMP-PD samples was performed using Illumina HiSeq X Ten sequencer, and data were processed against Human Genome Reference Build 38 (https://ftp.1000genomes.ebi.ac.uk/vol1/ftp/technical/reference/GRCh38_reference_genome/). Data cleaning was performed using PLINK v1.9 (RRID:SCR_001757; https://www.cog-genomics.org/plink/1.9/)^31^ and PLINK v2.0 (https://www.cog-genomics.org/plink/2.0/). For quality control at the sample level, we excluded individuals from analysis if they had a low genotyping call rate (≤95%), excessive heterozygosity rates (>±0.15 F-statistic) or a mismatch between clinically reported and genetically determined sex by the X chromosome. We also excluded duplicate or related samples (kinship coefficient > 0.088). We removed individuals that were not of European ancestry by performing a principal component analysis from pruned genetic data of each cohort included in the analysis. We used Hapmap3 as the reference panel to derive ancestry groups. Individuals that deviated by more than 3 standard deviations from the mean of the first two principal components of the HapMap3 CEU group were removed from the analysis.

For quality control at the variant level, we removed SNPs from analysis if they had a low genotyping rate (<0.99%), deviated significantly from the Hardy–Weinberg equilibrium (*P* < 1E^−8^), had a minor allele frequency <1% and were non-autosomal (X, Y, mitochondrial chromosomes). After quality control, genetic data for TPD and OPDC were imputed separately against the TOPMed (https://imputation.biodatacatalyst.nhlbi.nih.gov/#!)^32^  *r*2 panel with Eagle v2.4 phasing on the TOPMed Imputation Server using Minimac4.^33,34^ We used the Rsq info measure of imputation accuracy to exclude variants that were not confidently imputed. We filtered out variants with an Rsq lower than 0.8. We also removed SNPs if missingness was >5%, and minor allele frequency was <1%. The two data sets were then merged, with only shared variants retained.

### Statistical analysis for single-variant associations

Clinical data were cleaned and analysed using R v4.1.3 (RRID:SCR_001905; R Project for Statistical Computing, version 4.1.3; https://www.R-project.org/). We used logistic regression in PLINK to perform two separate genome-wide association studies for LBD-D (dementia with Lewy bodies and Parkinson’s disease dementia) compared with LBD-ND (Parkinson’s disease cases without dementia) in AMP-PD and in the merged TPD/OPDC data sets, respectively. The following covariates were incorporated in our model: age at onset (for TPD/OPDC cohorts) or age at diagnosis (for AMP-PD), sex, study and the first five genetic principal components. We meta-analysed the summary results for TPD/OPDC and AMP-PD using METAL (RRID:SCR_002013; http://csg.sph.umich.edu//abecasis/Metal/)^35^ under a random effects model using genomic control correction. We only included variants present in all cohorts and with a minor allele frequency variability below 15% across studies. We used Cochran’s Q to test for heterogeneity in the meta-analysis and excluded variants with *P*-value < 0.05 and *I*^2^ statistic≤ 80%. We considered *P*-values below 5 × 10^−8^ to be genome-wide significant and nominally significant below 5 × 10^−6^. We used LocusZoom to generate the Manhattan plot and the regional association plots (RRID:SCR_021374; http://locuszoom.org/).^36^

### Conditional analysis

In order to determine whether there were single or multiple independent signals at each genome-wide significant locus, we carried out a conditional and joint multiple-SNP analysis (COJO) on the GWAS summary statistics. We used the AMP-PD cohort as the reference panel to estimate the LD between the SNPs and apply corrections to the models as it is the largest participating cohort in the meta-analysis. COJO was performed using GCTA (v1.93.0, GCTA |  Yang Lab).^37^

### Colocalization analysis

We performed a colocalization analysis to investigate whether there is a shared causal variant between the risk of dementia in Lewy body disease cases and expression quantitative trait loci (eQTLs). We used the coloc R package (version 5.1.0; https://cran.rproject.org/web/packages/colocr/index.html)^38^ and colochelpR as a wrapper (version 0.99.0).^39^ coloc is based on a Bayesian statistical approach to compute a posterior probability (PP) for the following hypotheses: there is no association with either trait (H0); there is an association with the Lewy body dementia trait, but not the eQTL trait (H1); there is an association with the eQTL trait, but not the Lewy body dementia trait (H2); there is an association with a Lewy body dementia and an eQTL variant, but the causal variants are independent (H3); and there is a shared causal variant associated with Lewy body dementia and eQTL within the analysed region (H4). coloc was run using default per SNP priors p1 = 10–4, p2 = 10–4 and p12 = 10–5. A PPH4 > 0.80 was considered a statistically significant support for colocalization. We used Cis-eQTL data from eQTLGen, which include 31 684 individuals (https://www.eqtlgen.org/cis-eqtls.html) and compare genetic variation with blood RNA and PsychEncode. PsychEncode includes 1387 individuals (http://resource.psychencode.org/) and compares genetic variation with brain RNA. We extracted all the genes from ±1 Mb of the significant hits from the GWAS and performed a colocalization analysis on each gene. Since the cis-eQTL and the GWAS summary statistics were in different builds, we converted the summary statistics of the meta-analysis from hg38 to hg19 using the LiftOver tool (RRID:SCR_018160; https://genome.sph.umich.edu/wiki/LiftOver).

### Polygenic risk score

To assess the genetic overlap between LBD-D and Parkinson’s disease, dementia with Lewy bodies and Alzheimer’s disease risk profile, we computed a polygenic risk score (PRS) on all the LBD-D cases and LBD-ND for comparison. We used previously published Parkinson’s disease, dementia with Lewy bodies and Alzheimer’s disease GWAS^10,11,40^ as the reference data. After performing QC on summary statistics of the base data sets, we used PRSice-2 (version 2.3.5; RRID:SCR_017057; https://choishingwan.github.io/PRSice/)^41^ to calculate the PRS with the C + T method, which involves clumping SNPs and performing *P*-value thresholding. After clumping, 1 284 510 SNPs were included to generate the Parkinson’s disease PRS, 380 274 SNPs for the dementia with Lewy bodies PRS and 11 931 for the Alzheimer’s disease PRS. We then conducted general linear regression adjusted for age at onset, sex and PC1–PC5 to test if the PRS predicted the development of dementia. Results from the regression were meta-analysed in R with the meta package (RRID:SCR_019055; https://cran.r-project.org/web/packages/meta/index.html).

## Results

After QC, a total of 7804 individuals were selected, including 2908 LBD-D (2552 dementia with Lewy bodies, 357 Parkinson’s disease dementia) and 4896 LBD-ND. Case selection is summarized in Supplementary Fig. 1. Demographic characteristics are summarized in Table 1. LBD-D patients were significantly older than LBD-ND at diagnosis (Kruskal–Wallis Chi-squared = 2627, df = 1, *P*-value < 2.2e^−16^). There are more men than women in our study, and they are more likely to have a dementia phenotype (*P*-value = 3.09e^−06^). We determined that with the sample size we had, we were very well-powered (100% power) to detect genetic variants associated with dementia, assuming an odds ratio of 1.4 and a minor allele frequency of 0.15 under an additive model (see Supplementary Fig. 2).

### Identification of risk loci for dementia in Lewy body diseases

**Figure 1:**
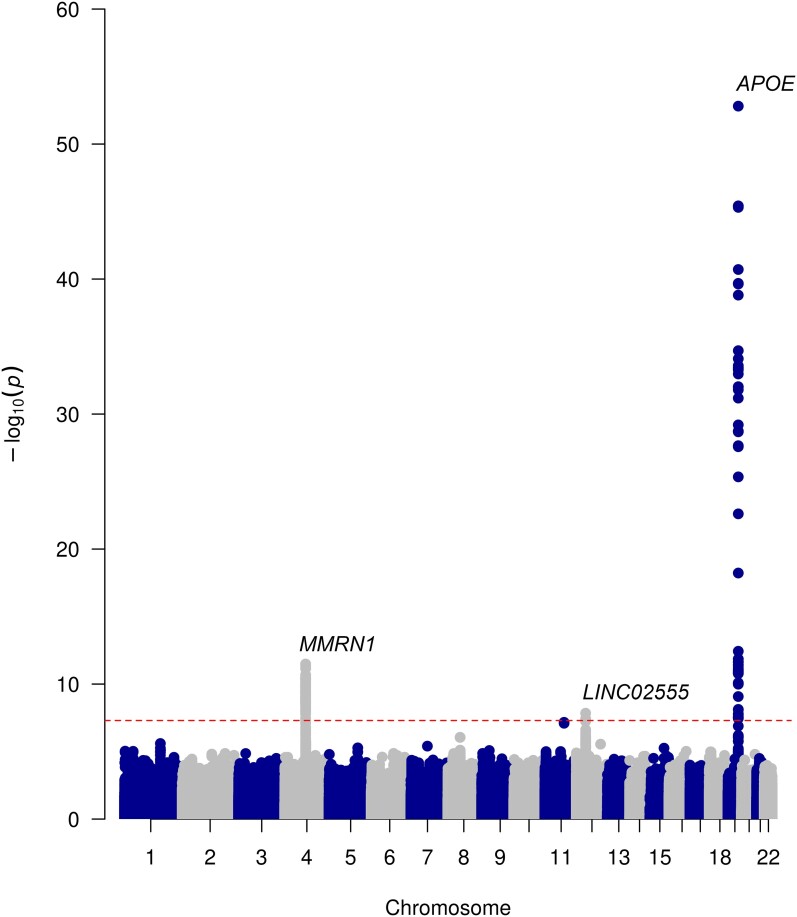
**Manhattan plot of LBD-D versus LBD-ND.** A Manhattan plot representing the results of the case–case genome-wide association study results (*n* = 2908 Lewy body disorders with dementia and *n* = 4896 Lewy body disorders without dementia), where 6 877 765 variants have been analysed under a logistic regression model. The plot highlights genome-wide significant single nuclear variants on chromosome 4 (rs7668531, *P* = 3.25e^−12^), 12 (rs17442721, *P* = 1.44e^−08^) and 19 (rs429358, *P* = 3.25e^−57^). Negative logarithm *P*-value is represented on the *y*-axis, while chromosome position is represented on the *x*-axis. The dotted line indicates genome-wide significant threshold (5 × 10^−8^).

Using a case–case GWAS approach comparing patients with LBD-D and LBD-ND, we analysed 6 226 081 SNPs and identified three genome-wide significant loci (Fig. 1, Table 2).

**Table 2:** Top SNPs from meta-analysis

CHR	BP	SNP ID	Effect allele	Nearest gene	Effect allele frequency					OR	CI	*P*-value
					Tracking Parkinson's and Oxford Discovery		AMP-PD		gnomAD			
					LBD-D	LBD-ND	LBD-D	LBD-ND	HC			
19	44908684	rs429358	C	APOE	0.2	0.13	0.27	0.13	0.15	2.606	2.307–2.943	3.25E^−57^
4	89870668	rs7668531	G	MMRN1	0.43	0.47	0.43	0.49	0.52	0.719	0.656–0.789	3.25E^−12^
12	40141971	rs17442721	G	LINC02555	0.01	0.02	0.02	0.07	0.02	0.427	0.318–0.573	1.44E^−08^
11	82697450	rs11233271	G	MIR4300HG	0.1	0.12	0.11	0.13	0.12	1.482	1.284–1.709	6.78E^−08^

Independent lead SNPs identified by LocusZoom. Genome coordinates are in build GRCh38. Allele frequency in European (non-Finnish) general population extracted from the gnomAD database (https://gnomad.broadinstitute.org/).

BP, base pair; chr, chromosome; CI, confidence interval; HC, healthy controls; OR, odds ratio; SNP, single nucleotide polymorphism.

The lead SNP was rs429358 in the *APOE* gene on chromosome 19 (OR = 2.606, 95% CI = 2.307–2.943, *P* = 3.25e^−57^; Supplementary Fig. 3A). *APOE* encodes apolipoprotein E, a known genetic factor for Alzheimer’s disease and dementia with Lewy bodies. It has also been identified as a risk factor for dementia in Parkinson’s disease.^16,42^ Conditional analysis on the lead SNP detected a secondary independent signal at the *APOE* locus at 19:32848205.

The second genome-wide significant SNP was rs7668531, an intergenic SNP between the *MMRN1* gene and the *SNCA-AS1* gene (OR = 0.719, 95% CI = 0.656–0.789, *P* = 3.25e^−12^; Supplementary Fig. 3B) located 170 323 kb downstream of the *SNCA* gene. This SNP is close to and in linkage disequilibrium with rs7680557 (*D*′ = 0.9959, *r*2 = 0.9196), which is associated with dementia as identified in the most recent Lewy body disease case–control GWAS.^11^ The rs7668531 signal is no longer genome-wide significant when we condition on the top rs7680557 in our data set, which suggests that rs7668531 is not independent and most likely tags *SNCA-AS1*.

The third genome-wide significant SNP was rs17442721 in the noncoding RNA *LINC02555*, which was protective against developing dementia (OR = 0.427, 95% CI = 0.318–0.573, *P* = 1.44e^−08^; Supplementary Fig. 3C). *LINC02555* is potentially a regulatory locus for *LRRK2* expression in specific cell types^43^ and may mediate PSP survival.^44^ However, this SNP is in LD with *LRRK2 G2019S* (rs34637584, *r*2 = 0.54, *D*′ = 0.97). To confirm whether rs17442721 is independent of *LRRK2 G2019S*, we performed a conditional analysis. Results show that rs17442721 is no longer genome-wide significant after conditioning on the G2019S variant, confirming that it tags *LRRK2* G2019S, and there is no difference in dementia related to this SNP when the data are stratified by G2019S status (Supplementary Table 2). In this data set, the rate of dementia in *LRRK2* G2019S carriers is 5% as compared with 39% in the total data set (Supplementary Table 3). rs17442721 was not in a linkage disequilibrium with PSP progression variant rs2242367 (*r*2 < 0.05).

Rs11233271 on chromosome 11 near the MIR4300HG gene approached genome-wide significance (OR = 1.48, 95% CI = 1.28–1.71, *P* = 6.78e^−08^), although this will need further evaluation in future work.

Common variant *GBA* E326K was nominally, but not genome-wide significant (OR = 2.01, 95% CI = 1.44–2.83, *P* = 2.517e^−06^). The Parkinson’s disease case–control GWAS *LRRK2* rs76904798 variant was also not genome-wide significant (OR = 1.02, 95% CI = 0.88–1.18, *P* = 0.7759).

### Colocalization

**Figure 2:**
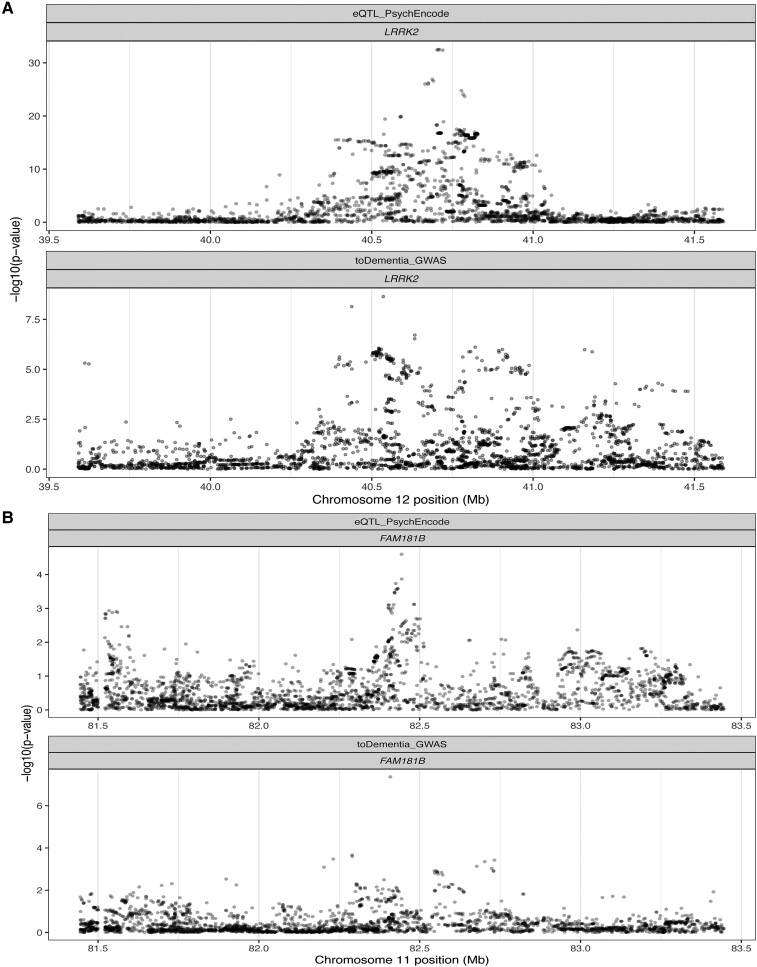
**Regional association plot for eQTL and GWAS signals.** Results from colocalization analysis presented via regional association plot for expression quantitative trait loci and (**A**) genome-wide association signals in the region close to LRRK2 (posterior probability H4 = 0.72, 4026 variants analysed) and (**B**) in the region close to FAM181B (posterior probability H4 = 0.70, 4357 variants analysed). Negative logarithm *P*-value is represented on the *y*-axis, while chromosome position is represented on the *x*-axis.

We performed a colocalization analysis to assess the probability of a shared causal signal between dementia status and genetically determined gene expression regulation. eQTLs were obtained from eQTLGen and PsychENCODE. eQTLGen comprises gene expression derived from blood, and psychENCODE data set comprises gene expression from bulk RNA sequencing from the frontal cortex. We found a suggestive colocalization between the genome-wide significant signal on chromosome 12 and cis-eQTL data from eQTLGen (PPH4 = 0.7154) for *LRRK2,* and rs11233271 on chromosome 11 suggestively colocalized with *FAM181B* (PPH4 = 0.7009), a protein-coding gene that is expressed in the brain (Fig. 2).

### Polygenic risk score

**Figure 3:**
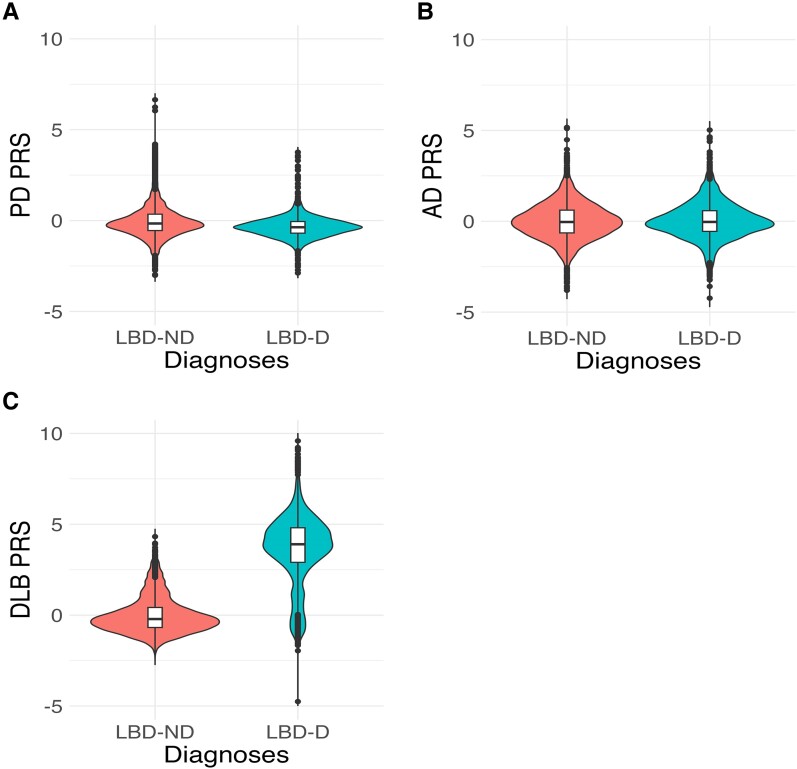
**Polygenic risk score from Parkinson’s disease, Alzheimer’s disease and dementia with Lewy bodies GWAS.** Violin plot comparing z-transformed (**A**) Parkinson's disease, (**B**) Alzheimer's disease and (**C**) dementia with Lewy body polygenic risk score (PRS) distributions in Lewy body disease with dementia (LBD-D, *n* = 2908) with those without (LBD-ND, *n* = 4896). The centreline of the box plot represents the median, and the box limits are the interquartile range. Dots correspond to outliers. A general linear regression model was applied to assess the odds PRS-predicted dementia. High Parkinson’s disease PRS predicts lower odds of developing dementia (OR = 0.74, 95% CI = 0.56–0.98, *P* = 0.03), while high dementia with Lewy bodies PRS predicts increased odds of developing dementia (OR = 2.69, 95% CI = 0.69–10.42, *P* = 0.01).

We applied a Parkinson’s disease, Alzheimer’s disease and dementia with Lewy bodies PRS derived from the most recent GWAS to the LBD-D patients identified in each of our cohorts, as well as to LBD-ND patients for comparison. We used a general linear regression model to assess if the normalized PRS predicted dementia and meta-analysed the regressions using a random effects model. Optimized *P*-value PRS based on genome-wide significant and sub-genome significant SNPs indicated that patients with a higher Parkinson’s disease PRS score (based on 1 284 510 SNPs) were less likely to develop dementia (OR = 0.74, 95% CI = 0.56–0.98, *P* = 0.03), and the Alzheimer’s disease risk profile (based on 31 000 SNPs) was not significantly different between the two groups (OR = 0.99, 95% CI = 0.82–1.20, *P* = 0.93). LBD-D was significantly associated with higher (pure) dementia with Lewy bodies PRS (OR = 2.69, 95% CI = 0.69–10.42, *P* = 0.01); however, this needs to be interpreted with caution as the confidence interval is very large (Fig. 3).

## Discussion

We have conducted a large-scale genome-wide case–case analysis to understand the genetic drivers of dementia in Lewy body diseases by comparing Lewy body diseases with and without dementia and identified three independent genome-wide significant signals in a novel case–case analysis by comparing LB cases with dementia with cases unaffected by dementia.

In line with previous studies, we showed that *APOE e4* is the strongest risk factor for dementia in Lewy body diseases. Given the role of *APOE e4* in Alzheimer’s disease, this may modulate the risk of dementia via Alzheimer’s disease pathology in at least a subset of the LBD-D cases; however, previous work has been inconsistent. A substantial proportion (30–40%) of patients with Parkinson’s disease and 50–80% of patients with dementia with Lewy bodies have co-occurring Alzheimer’s disease pathology.^45^ However, it is unclear whether *APOE e4* drives dementia via Alzheimer’s disease pathology or independently. Our results indicate that the Alzheimer’s disease PRS does not drive dementia in Lewy body diseases, suggesting that *APOE e4* may drive dementia in these cases by an Alzheimer’s disease pathology-independent mechanism. Consistent with our findings, postmortem studies have found that *APOE e4* was associated with dementia in Lewy body diseases in both ‘pure’ Lewy body diseases and those with Alzheimer’s disease co-pathology.^25^ It is also possible that *APOE e4* mediates neurodegenerative processes via neuroinflammation independently of amyloid and tau pathology.^46,47^ In fact, inflammation markers are apparent before protein aggregation.^47^ For instance, a longitudinal study showed that blood–brain barrier dysfunction at baseline predicted future cognitive decline in *APOE4* carriers, but not in non-carriers.^48^ Further research is needed to clarify the role of *APOE e4* in the Lewy body disease pathology.

We also found a SNP between *MMRN1* and the 5′ end of *SNCA*, but not at the 3′ end to be significantly associated with lower odds of developing dementia, consistent with previous candidate gene studies^13^ and GWAS.^11^ Postmortem studies have found that alpha-synuclein in cortical areas is a predictor of dementia in Lewy body diseases. The finding that *SNCA-AS1* is specific to LBD-D makes it an interesting potential therapeutic target. Indeed, LBD-D tends to have a much more aggressive disease course with faster progression to mortality. Targeting *SNCA-AS1* could therefore be a potential solution to reducing the alpha-synuclein pathology in the cortex and the progression to dementia in Lewy body diseases. Our study has separated the 3′ signal in SNCA, which is associated with Parkinson’s disease risk, from the 5′ signal associated with dementia in Lewy body diseases. We hypothesize that the 3′ signal is important for the level of *SNCA* expression and the initiation of the Parkinson’s disease process, particularly in subcortical areas, whereas the 5′ SNP is associated with the expression of *SNCA* in the cortex.^49^ Indirectly, this suggests that local *SNCA* expression is important, distinct from cell to cell spread from subcortical areas.

The third genome-wide significant signal was located near *LINC02555*, which is potentially a regulator of *LRRK2*. However, we confirmed that this SNP is tagging *LRRK2* G2019S. In the present study, we did not exclude *LRRK2* mutation carriers from the main analysis. As previously described, we have shown in this study that *LRRK2* G2019S carriers are less likely to develop dementia.^50^ Moreover, *LRRK2* likely does not play a major role in dementia with Lewy bodies.^51^ Our results confirm that in Lewy body diseases, the *LRRK2* G2019S mutation status is associated with decreased odds of progression towards dementia.

Rs11233271 on chromosome 11 close to MIR4300HG was nominally significant in our GWAS. This SNP may regulate the expression of *FAM181B*, a protein-coding gene involved in the development of the nervous system.^52^  *FAM181B* was also associated with working memory in a gene-based study on cognitive measures in adolescence.^53^ Furthermore, this locus has been associated with variation in the microbiome. Further studies are needed to investigate the role of this locus in Lewy body dementia.

Interestingly, *GBA1*, *BIN1* and *TMEM175*, which are associated with case–control Lewy body disease GWAS^11^, did not appear significant when comparing LBD-D with LBD-ND. Since *GBA1* is a known risk gene for both Parkinson’s disease and dementia with Lewy bodies, our analysis shows that variation in *GBA1* does not distinguish between LBD-D and LBD-ND within a study of this size. Similarly, *TMEM175* is a risk factor in both Lewy body diseases with and without dementia. Therefore, it is not surprising that the signal disappears when we make a head-to-head comparison. *BIN1* encodes bridging integrator 1 and is the second strongest signal associated with Alzheimer’s disease, but was not genome-wide significantly associated with LBD-D in this study (*P* = 2.276e^−05^).^54^ Increased *BIN1* expression is associated with a higher load of tau in the Alzheimer’s disease brain, but not amyloid.^55^ While some studies found the tau load to be a correlate of dementia in Parkinson’s disease and dementia with Lewy bodies, other studies have not. Autopsy studies have found tau to colocalize with alpha-synuclein in Lewy bodies in both Parkinson’s disease and dementia with Lewy bodies.^56^ A small autopsy study in *LRRK2* carriers found that 100% of the brains had tau pathology.^57^ Therefore, it is possible that the Lewy body disease risk genes associated with tau pathology are not good candidates to distinguish LBD-D from LBD-ND. *RIMS2* was identified as a progression locus in a genome-wide survival study of Parkinson’s disease dementia^15^; however, this was not genome-wide significant in the present study (*P* = 0.016).

We acknowledge several limitations of our study. First, the analysis only included patients of European ancestry and is therefore not generalizable to other populations. As in previous studies, there are more men than women in our study.^58,59^ Our main results apply to men and women, but we have not carried out a sex stratified analysis to look for sex-specific loci associated with dementia. In addition, it is possible that some patients were censored as non-demented based on the clinical data available, but who might have developed dementia if followed-up for a longer period of time. We grouped patients who developed dementia at any time point together in the design of our study. However, it is possible that genetic risk factors and associated neuropathology leading to dementia at onset are different from those associated with dementia later in the disease course. We hypothesize that Parkinson’s disease patients developing dementia early in the disease course will be genetically closer to dementia with Lewy bodies, while those developing dementia much later on will present with a different genetic profile. Future studies should aim to identify risk factors leading to a more aggressive disease course in Lewy body diseases to improve prognosis and care.

In conclusion, in a pooled analysis of dementia with Lewy bodies, Parkinson’s disease and Parkinson’s disease dementia, we have shown that *APOE e4* is the major determinant of Lewy body diseases with dementia. We have also shown that variation at the 5′ end of the *SNCA* gene and variant tagging *LRRK2* G2019S are associated with a significantly reduced risk of dementia. Although *APOE* is associated with dementia, other Alzheimer’s disease risk loci defined by PRS analysis were not associated with LBD-dementia. Increasing sample sizes in collaborative international studies will help resolve the disease pathogenesis, the nosological overlap between Parkinson’s disease dementia and dementia with Lewy bodies, and ultimately help define new treatments.

## Supplementary Material

fcae190_Supplementary_Data

## Data Availability

TPD data are available upon access request from https://www.dpag.ox.ac.uk/opdc/team/proband-tracking-parkinsons. AMP-PD data are available upon registration at https://www.amp-pd.org/. OPDC data are available upon request from the Dementias Platform UK (https://portal.dementiasplatform.uk/Apply). HapMap phase 3 data (HapMap3) are available for download at https://www.broadinstitute.org/medical-and-population-genetics/hapmap-3. Cis-QTL eQTLGen data were downloaded from (https://www.eqtlgen.org/cis-eqtls.html). eQTL data from eQTL catalogue can be ftp-accessed (https://www.ebi.ac.uk/eqtl/Data_access/). Summary statistics from the Parkinson’s disease GWAS (Nalls *et al.*)^10^ used to perform the PRS analysis are available from https://pdgenetics.org/resources. The source code is available on GitHub (https://github.com/huw-morris-lab/LBD-case-case-GWAS; https://doi.org/10.5281/zenodo.8335404).

## Appendix

### International LBD Genomics Consortium (iLBDGC)

#### United States

- **Yevgeniya Abramzon, B.Sc.** (Neuromuscular Diseases Research Section, Laboratory of Neurogenetics, National Institute on Aging, Bethesda, MD, 20892, USA)
- **Sarah Ahmed, B.Sc.** (Neurodegenerative Diseases Research Unit, Laboratory of Neurogenetics, National Institute of Neurological Disorders and Stroke, Bethesda, MD, 20892, USA)
- **Camille Alba, Ph.D.** (Department of Anatomy, Physiology and Genetics, Physiology and Genetics, Uniformed Services University of the Health Sciences, Bethesda, MD, 20814, USA)
- **Marilyn S. Albert, Ph.D.** (Department of Neurology, Johns Hopkins University Medical Center, Baltimore, MD, 21287, USA)
- **Dagmar Bacikova, Ph.D.** (Department of Anatomy, Physiology and Genetics, Physiology and Genetics, Uniformed Services University of the Health Sciences, Bethesda, MD, 20814, USA)
- **Matthew J. Barrett, M.D.** (Department of Neurology, University of Virginia School of Medicine, Charlottesville, VA, 22903, USA)
- **Thomas G. Beach, M.D., Ph.D.** (Civin Laboratory for Neuropathology, Banner Sun Health Research Institute, Sun City, AZ, 85006, USA)
- **David A. Bennett, M.D.** (Rush Alzheimer's Disease Center, Rush University, Chicago, IL, 60612, USA)
- **Lilah M. Besser, Ph.D., M.Ph.** (Institute for Human Health and Disease Intervention, Florida Atlantic University, Boca Raton, FL, 33431, USA)
- **Eileen H. Bigio, M.D.** (Mesulam Center for Cognitive Neurology and Alzheimer's Disease, Northwestern University Feinberg School of Medicine, Chicago, IL, 60611, USA)
- **Bradley F. Boeve, M.D.** (Center for Sleep Medicine, Mayo Clinic, Rochester, MN, 55905, USA)
- **Ryan C. Bohannan, B.Sc.** (Department of Neurobiology and Behavior, University of California Irvine, Irvine, CA, 92697, USA)
- **Chad A. Caraway, Ph.D.** (Institute for Memory Impairments and Neurological Disorders, University of California Irvine, Irvine, CA, 92697, USA)
- **Jose-Alberto Palma, M.D., Ph.D.** (Department of Neurology, New York University School of Medicine, New York, NY, 10016, USA)
- **Ruth Chia, Ph.D.** (Neuromuscular Diseases Research Section, Laboratory of Neurogenetics, National Institute on Aging, Bethesda, MD, 20892, USA)
- **Clifton L. Dalgard, Ph.D.** (Department of Anatomy, Physiology and Genetics, Uniformed Services University of the Health Sciences, Bethesda, MD, 20814, USA; The American Genome Center, Collaborative Health Initiative Research Program, Uniformed Services University of the Health Sciences, Bethesda, MD, 20814, USA)
- **Dennis Dickson, M.D.** (Department of Neuroscience, Mayo Clinic, Jacksonville, FL, 32224, USA)
- **Jinhui Ding, Ph.D.** (Computational Biology Core, Laboratory of Neurogenetics, National Institute on Aging, Bethesda, MD, 20892, USA)
- **Kelley Faber, M.Sc.** (Department of Medical and Molecular Genetics, Indiana University School of Medicine, Indianapolis, IN, 46202, USA)
- **Tanis Ferman, Ph.D.** (Department of Psychiatry and Psychology, Mayo Clinic, Jacksonville, FL, 32224, USA)
- **Luigi Ferrucci, M.D., Ph.D.** (Longitudinal Studies Section, National Institute on Aging, Baltimore, MD, 21224, USA)
- **Margaret E. Flanagan, M.D.** (Northwestern University Feinberg School of Medicine, Chicago, IL, 60611, USA)
- **Tatiana M. Foroud, M.D.** (Department of Medical and Molecular Genetics, Indiana University School of Medicine, Indianapolis, IN, 46202, USA)
- **Bernardino Ghetti, M.D.** (Department of Pathology and Laboratory Medicine, Indiana University School of Medicine, Indianapolis, IN, 46202, USA)
- **J. Raphael Gibbs, Ph.D.** (Computational Biology Core, Laboratory of Neurogenetics, National Institute on Aging, Bethesda, MD, 20892, USA)
- **Alison Goate, Ph.D.** (Nash Family Department of Neuroscience, Department of Genetics and Genomic Sciences, and Department of Pathology, Icahn School of Medicine at Mount Sinai, New York, NY, 10029, USA)
- **David Goldstein, M.D.** (Clinical Neurocardiology Section, National Institute of Neurological Disorders and Stroke, Bethesda, MD, 20892, USA)
- **Neill R. Graff-Radford, M.D.** (Department of Neurology, Mayo Clinic Florida, 4500 San Pablo Road South, Jacksonville, FL, 32224, USA)
- **Heng-Chen Hu, Ph.D.** (Department of Anatomy, Physiology and Genetics, Physiology and Genetics, Uniformed Services University of the Health Sciences, Bethesda, MD, 20814, USA)
- **Daniel Hupalo, Ph.D.** (Department of Anatomy, Physiology and Genetics, Physiology and Genetics, Uniformed Services University of the Health Sciences, Bethesda, MD, 20814, USA)
- **Scott M. Kaiser, M.B.A.** (Department of Neuropathology, Indiana University School of Medicine, Indianapolis, IN, 46202, USA)
- **Horacio Kaufmann, M.D.** (Department of Neurology, New York University School of Medicine, New York, NY, 10016, USA)
- **Ronald C. Kim, M.D.** (Department of Neuropathology, School of Medicine, University of California Irvine, Irvine, CA, 92697, USA)
- **Gregory Klein** (Rush Alzheimer's Disease Center, Rush University, Chicago, IL, 60612, USA)
- **Walter Kukull, Ph.D.** (National Alzheimer's Coordinating Center (NACC), University of Washington, Seattle, WA, 98195, USA)
- **Amanda Kuzma, M.Sc.** (Department of Pathology and Laboratory Medicine, Perelman School of Medicine, University of Pennsylvania, Philadelphia, PA, USA)
- **James Leverenz, M.D.** (Cleveland Lou Ruvo Center for Brain Health, Neurological Institute, Cleveland Clinic, Cleveland, OH, 44195, USA)
- **Grisel Lopez, M.D.** (Medical Genetics Branch, National Human Genome Research Institute, Bethesda, MD, 20892, USA)
- **Qinwen Mao, M.D., Ph.D.** (Northwestern University Feinberg School of Medicine, Chicago, IL, 60611, USA)
- **Elisa Martinez-McGrath, Ph.D.** (Department of Anatomy, Physiology and Genetics, Physiology and Genetics, Uniformed Services University of the Health Sciences, Bethesda, MD, 20814, USA)
- **Eliezer Masliah, M.D.** (Molecular Neuropathology Section, Laboratory of Neurogenetics, National Institute on Aging, Bethesda, MD, 20892, USA)
- **Ed Monuki, M.D., Ph.D.** (Department of Pathology & Laboratory Medicine, School of Medicine, University of California Irvine, Irvine, CA, 92697, USA)
- **Kathy L. Newell, M.D.** (Department of Pathology and Laboratory Medicine, University of Kansas Medical Center, Kansas City, KS, 66160, USA)
- **Lucy Norcliffe-Kaufmann, Ph.D.** (Department of Neurology, New York University School of Medicine, New York, NY, 10016, USA)
- **Matthew Perkins, B.Sc.** (Michigan Brain Bank, University of Michigan Medical School, Ann Arbor, MI, 48109, USA)
- **Olga Pletnikova, M.D.** (Department of Pathology [Neuropathology], Johns Hopkins University Medical Center, Baltimore, MD, 21287, USA)
- **Alan E. Renton, Ph.D.** (Department of Neuroscience, Icahn School of Medicine at Mount Sinai, New York, NY, 10029, USA)
- **Susan M. Resnick, M.D.** (Laboratory of Behavioral Neuroscience, National Institute on Aging, Baltimore, MD, 21224, USA)
- **Owen A. Ross, Ph.D.** (Department of Neuroscience & Department of Clinical Genomics, Mayo Clinic Florida, 4500 San Pablo Road South, Jacksonville, FL, 32224, USA)
- **Marya S. Sabir, B.Sc.** (Neurodegenerative Diseases Research Unit, Laboratory of Neurogenetics, National Institute of Neurological Disorders and Stroke, Bethesda, MD, 20892, USA)
- **Clemens R. Scherzer, M.D.** (Precision Neurology Program, Brigham & Women's Hospital, Harvard Medical School, Boston, MA, 02115, USA)
- **Sonja W. Scholz, M.D., Ph.D.** (Neurodegenerative Diseases Research Unit, Laboratory of Neurogenetics, National Institute of Neurological Disorders and Stroke, Bethesda, MD, 20892, USA; Department of Neurology, Johns Hopkins University Medical Center, Baltimore, MD, 21287, USA)
- **Geidy Serrano, Ph.D.** (Civin Laboratory for Neuropathology, Banner Sun Health Research Institute, Sun City, AZ, 85006, USA)
- **Vikram Shakkotai, M.D., Ph.D.** (Department of Neurology, University of Michigan Medical School, Ann Arbor, MI, 48109, USA)
- **Ellen Sidransky, M.D.** (Medical Genetics Branch, National Human Genome Research Institute, Bethesda, MD, 20892, USA)
- **Andrew B. Singleton, Ph.D.** (Molecular Genetics Section, Laboratory of Neurogenetics, National Institute on Aging, Bethesda, MD, 20892, USA)
- **Toshiko Tanaka, Ph.D.** (Longitudinal Studies Section, National Institute on Aging, Baltimore, MD, 21224, USA)
- **Nahid Tayebi, Ph.D.** (Medical Genetics Branch, National Human Genome Research Institute, Bethesda, MD, 20892, USA)
- **Bryan J. Traynor, M.D., Ph.D.** (Neuromuscular Diseases Research Section, Laboratory of Neurogenetics, National Institute on Aging, Bethesda, MD, 20892, USA; Department of Neurology, Johns Hopkins University Medical Center, Baltimore, MD, 21287, USA)
- **Juan C. Troncoso, M.D.** (Department of Pathology [Neuropathology], Johns Hopkins University Medical Center, Baltimore, MD, 21287, USA)
- **Coralie Viollet, Ph.D.** (Department of Anatomy, Physiology and Genetics, Physiology and Genetics, Uniformed Services University of the Health Sciences, Bethesda, MD, 20814, USA)
- **Ronald L. Walton, B.Sc.** (Department of Neuroscience, Mayo Clinic Florida, Jacksonville, FL, 32224, USA)
- **Randy Woltjer, M.D., Ph.D.** (Department of Neurology, Oregon Health & Sciences University, Portland, OR, 97239, USA)
- **Zbigniew K. Wszolek, M.D.** (Department of Neurology, Mayo Clinic Florida, 4500 San Pablo Road South, Jacksonville, FL, 32224, USA)

#### Canada

- **Sandra E. Black, M.D.** (Institute of Medical Science, Faculty of Medicine, University of Toronto, 1 King's College Circle, Room 2374, Toronto, ON, M5S 1A8, Canada; Division of Neurology, Department of Medicine, University of Toronto, 27 King's College Circle, Toronto, ON, M5S 1A1, Canada; Heart and Stroke Foundation Canadian Partnership for Stroke Recovery, Sunnybrook Health Sciences Centre, University of Toronto, 1 King's College Circle, Room 2374, Toronto, ON, M5S 1A8, Canada; Hurvitz Brain Sciences Research Program, Sunnybrook Research Institute, University of Toronto, 2075 Bayview Avenue, Toronto, ON, M4N 3M5, Canada; LC Campbell Cognitive Neurology Research Unit, Sunnybrook Research Institute, University of Toronto, 2075 Bayview Avenue, Toronto, ON, M4N 3M5, Canada)
- **Ziv Gan-Or, M.D., Ph.D.** (Montreal Neurological Institute and Hospital, Department of Neurology & Neurosurgery, McGill University, 3801 University Street, Montreal, QC, H3A 2B4, Canada)
- **Julia Keith, M.D.** (Department of Anatomical Pathology, Sunnybrook Health Sciences Centre, University of Toronto, 1 King's College Circle, Room 2374, Toronto, ON, M5S 1A8, Canada)
- **Mario Masellis, M.D., Ph.D.** (Cognitive & Movement Disorders Clinic, Sunnybrook Health Sciences Centre, University of Toronto, 1 King's College Circle, Room 2374, Toronto, ON, M5S 1A8, Canada; Department of Medicine, Division of Neurology, University of Toronto, Toronto, ON, M5S 1A8, Canada; Hurvitz Brain Sciences Research Program, Sunnybrook Research Institute, University of Toronto, Toronto, ON, M5S 1A8, Canada; LC Campbell Cognitive Neurology Research Unit, Sunnybrook Research Institute, University of Toronto, Toronto, ON, M5S 1A8, Canada)
- **Ekaterina Rogaeva, Ph.D.** (Tanz Centre for Research in Neurodegenerative Diseases, University of Toronto, 1 King's College Circle, Room 2111, Toronto, ON, M5S 1A8, Canada)

#### United Kingdom

- **Dag Aarsland, M.D.** (Department of Old Age Psychiatry, Institute of Psychiatry, Psychology and Neuroscience [IoPPN], King's College London, DeCrespigny Park, London, SE5 8AF, UK)
- **Safa Al-Sarraj, M.D., Ph.D.** (Department of Clinical Neuropathology and London Neurodegenerative Diseases Brain Bank, Institute of Psychiatry, Psychology and Neuroscience [IoPPN], King's College Hospital and King's College London, DeCrespigny Park, London, SE5 8AF, UK)
- **Johannes Attems, M.D.** (Mental Health, Dementia and Neurodegeneration, Translational and Clinical Research Institute, Newcastle University, Newcastle upon Tyne, NE4 5PL, UK)
- **Raffaele Ferrari, Ph.D.** (Department of Molecular Neuroscience, Institute of Neurology, University College London, London, WC1B 5EH, UK)
- **Steve Gentleman, M.D.** (Neuropathology Unit, Division of Brain Sciences, Department of Medicine, Imperial College London, London, W12 0NN, UK)
- **John A. Hardy, Ph.D.** (Department of Neurodegenerative Disease, Reta Lila Weston Laboratories, Queen Square Genomics, UCL Dementia Research Institute, London WC1E 6BT, UK)
- **Angela K. Hodges, Ph.D.** (Department of Old Age Psychiatry, Institute of Psychiatry, Psychology and Neuroscience [IoPPN], King's College London, Maurice Wohl Clinical Neuroscience Institute, London, SE5 9NU, UK)
- **Seth Love, M.D.** (Dementia Research Group, School of Clinical Sciences, University of Bristol, Bristol, BS10 5NB, UK)
- **Ian McKeith, M.D.** (Mental Health, Dementia and Neurodegeneration, Translational and Clinical Research Institute, Newcastle University, Newcastle upon Tyne, NE4 5PL, UK)
- **Christopher M. Morris, Ph.D.** (Mental Health, Dementia and Neurodegeneration, Translational and Clinical Research Institute, Newcastle University, Newcastle upon Tyne, NE4 5PL, UK)
- **Huw R. Morris, M.D., Ph.D.** (Department of Molecular Neuroscience, Institute of Neurology, University College London, London, WC1B 5EH, UK; Department of Clinical and Movement Neuroscience, Royal Free Campus UCL Institute of Neurology, University College London, London, NW3 2PF, UK)
- **Laura Palmer, Ph.D.** (South West Dementia Brain Bank, Bristol Medical School, University of Bristol, Bristol, BS10 5NB, UK)
- **Stuart Pickering-Brown, Ph.D.** (Division of Neuroscience and Experimental Psychology, Faculty of Biology, Medicine and Health, The University of Manchester, Manchester, M13 9PT, UK)
- **Regina H. Reynolds, M.Sc.** (NIHR Great Ormond Street Hospital Biomedical Research Centre, University College London, London, UK; Genetics and Genomic Medicine, Great Ormond Street Institute of Child Health, University College London, London WC1E 6BT, UK; Department of Neurodegenerative Disease, UCL Queen Square Institute of Neurology, University College London, London, UK)
- **Mina Ryten, M.D., Ph.D.** (NIHR Great Ormond Street Hospital Biomedical Research Centre, University College London, London, UK; Genetics and Genomic Medicine, Great Ormond Street Institute of Child Health, University College London, London WC1E 6BT, UK)
- **Alan J. Thomas, M.B.B.S., Ph.D.** (Mental Health, Dementia and Neurodegeneration, Translational and Clinical Research Institute, Newcastle University, Newcastle upon Tyne, NE4 5PL, UK)
- **Bension S. Tilley, M.B.B.S., Ph.D.** (Neuropathology Unit, Division of Brain Sciences, Department of Medicine, Imperial College London, London, W12 0NN, UK)
- **Claire Troakes, Ph.D.** (Department of Old Age Psychiatry, Department of Basic and Clinical Neuroscience, King's College London, DeCrespigny Park, London, SE5 8AF, UK)

#### Republic of Ireland

- **Francesca Brett, M.D.** (Dublin Brain Bank, Neuropathology Department, Beaumont Hospital, Dublin, D2, Ireland)

#### France

- **Alexis Brice, M.D.** (Paris Brain Institute, Paris Brain Institute, Sorbonne Universites, 47, boulevard de l'Hôpital, Paris, CS 21 414 – 75646, France)
- **Charles Duyckaerts, M.D.** (Paris Brain Institute, Paris Brain Institute, Sorbonne Universites, 47, boulevard de l'Hôpital, Paris, CS 21 414 – 75646, France)
- **Suzanne Lesage, Ph.D.** (Paris Brain Institute, ICM, Sorbonne Universites, 47, boulevard de l'Hôpital, Paris, CS 21 414 – 75646, France)

#### Italy

- **Maura Brunetti, M.Sc.** (Rita Levi Montalcini Department of Neuroscience, University of Turin, Turin, 10126, Italy)
- **Andrea Calvo, M.D.** (Rita Levi Montalcini Department of Neuroscience, University of Turin, Turin, 10126, Italy)
- **Antonio Canosa, M.D.** (Rita Levi Montalcini Department of Neuroscience, University of Turin, Turin, 10126, Italy)
- **Adriano Chiò, M.D.** (Rita Levi Montalcini Department of Neuroscience, University of Turin, Turin, 10126, Italy; Institute of Cognitive Sciences and Technologies, C.N.R., Via S. Martino della Battaglia, 44, Rome, 00185, Italy, Azienda Ospedaliero Universitaria Città della Salute e della Scienza, Corso Bramante, 88, Turin, 10126, Italy)
- **Gianluca Floris, M.D.** (Department of Neurology, University Hospital of Cagliari, Via Ospedale, 54, Cagliari, 09124, Italy)
- **Giancarlo Logroscino, M.D., Ph.D.** (Department of Basic Medical Sciences, Neurosciences and Sense Organs, University of Bari “Aldo Moro”, Bari, 70121, Italy)
- **Chiara Zecca, B.Sc.** (Department of Basic Medical Sciences, Neurosciences and Sense Organs, University of Bari “Aldo Moro”, Bari, 70121, Italy)
- **Jordi Clarimon, Ph.D.** (Universitat Autònoma de Barcelona, Carrer de Sant Quintí, 77-79, Barcelona, 08041, Spain, and Movement Disorders Units, Department of Neurology, University Hospital Mutua de Terrassa, Barcelona, 08221, Spain)
- **Monica Diez-Fairen, M.Sc.** (Memory and Movement Disorders Units, Department of Neurology, University Hospital Mutua de Terrassa, 08221 Barcelona, Spain)
- **Juan Fortea, M.D., Ph.D.** (Universitat Autònoma de Barcelona, Carrer de Sant Quintí, 77-79, Barcelona, 08041, Spain, and Movement Disorders Units, Department of Neurology, University Hospital Mutua de Terrassa, Barcelona, 08221, Spain)
- **Isabel González-Aramburu, M.D., Ph.D.** (Neurology Service, University Hospital Marqués de Valdecilla-IDIVAL-UC, Santander, 39011, Spain)
- **Jon Infante, M.D., Ph.D.** (Neurology Service, University Hospital Marqués de Valdecilla-IDIVAL-UC, Santander, 39011, Spain)
- **Carmen Lage, M.D.** (Neurology Service, University Hospital Marqués de Valdecilla-IDIVAL-UC, Santander, 39011, Spain)
- **Alberto Lleó, M.D., Ph.D.** (Universitat Autònoma de Barcelona, Carrer de Sant Quintí, 77-79, Barcelona, 08041, Spain, and Movement Disorders Units, Department of Neurology, University Hospital Mutua de Terrassa, Barcelona, 08221, Spain)
- **Pau Pastor, M.D., Ph.D.** (Memory and Movement Disorders Units, Department of Neurology, University Hospital Mutua de Terrassa, 08221 Barcelona, Spain)
- **Laura Porcel-Molina, M.D., Ph.D.** (Neurological Tissue Bank, Biobanc-Hospital Clinic - IDIBAPS, C/ Centre Esther Koplowitz, Barcelona, 08036, Spain)
- **Eloy Rodríguez-Rodríguez, M.D., Ph.D.** (Neurology Service, University Hospital Marqués de Valdecilla-IDIVAL-UC, Santander, 39011, Spain)
- **Pascual Sanchez-Juan, M.D., Ph.D.** (Neurology Service, University Hospital Marqués de Valdecilla-IDIVAL-UC, Santander, 39011, Spain)

#### Luxembourg

- **Rejko Krüger, M.D.** (Luxembourg Center for Systems Biomedicine, University of Luxembourg, Esch-sur-Alzette, Luxembourg, L-4362, Luxembourg; Luxembourg Institute of Health (LIH), University of Luxembourg, Esch-sur-Alzette, Luxembourg, L-4362, Luxembourg; Centre Hospitalier de Luxembourg (CHL), University of Luxembourg, Esch-sur-Alzette, Luxembourg, L-4362, Luxembourg)
- **Patrick May, Ph.D.** (Luxembourg Center for Systems Biomedicine, University of Luxembourg, Esch-sur-Alzette, Luxembourg, L-4362, Luxembourg)

#### Greece

- **Georgia Xiromerisiou, M.D., Ph.D.** (Department of Neurology, University Hospital of Larissa, University of Thessalia, Mezourlo, Larissa, 41110, Greece)
