# Supplementary material for: Investigation of the genetic aetiology of Lewy body diseases with and without dementia
Source: Brain Commun. 2024 May 31;6(4):fcae190. doi: 10.1093/braincomms/fcae190 (PMC11228432; doi:10.1093/braincomms/fcae190)
Supplement: fcae190_Supplementary_Data [file fcae190_supplementary_data.pdf]

Supplementary Table 1. Cohort description

Cohort	Abbreviation	N	Genotyping array
Tracking Parkinson's Disease	TPD	2000	Illumina HumanCoreExome array
Oxford Parkinson's Disease Centre Discovery Cohort	OPDC	1082	Illumina HumanCoreExome-12 v1.1 or Illumina Infinium HumanCoreExome-24 v1.1
BioFIND	BF	213	Whole Genome Sequenced
Harvard Biomarker Study	HB	1173	Whole Genome Sequenced
Lewy Body Dementia Study	LB	4579	Whole Genome Sequenced
LRRK2 Cohort Consortium	LC	599	Whole Genome Sequenced
Parkinson's Disease Biomarkers Program	PD	1604	Whole Genome Sequenced
Parkinson's Progression Markers Initiative	PP	1943	Whole Genome Sequenced
Steady PD - Phase 3	SY	329	Whole Genome Sequenced
Sure PD - Phase 3	SU	259	Whole Genome Sequenced

Supplementary Table 2. Allele frequency of top SNP on chromosome 12 and *LRRK2* G2019S tagging SNP

Groups		Rs17442721	Rs34637584 (*LRRK2* G2019S)
LRRK2 carriers	LBD	45%	45%
	Non LBD	47%	46%
Non LRRK2 carriers	LBD	2%	0%
	Controls*	2%	0%

*Data extracted from non-Finnish Europeans in gnomAD browser (https://gnomad.broadinstitute.org/variant/12-40141971-C-G?dataset=gnomad_r3)

Supplementary Table 3. Dementia in *LRRK2* G20219S carriers

Groups	LBD-D	LBD-ND
Patients without *LRRK2 G2019S*	2893 (39%)	4593 (61%)
Patients with *LRRK2 G2019S*	15 (5%)	303 (95%)

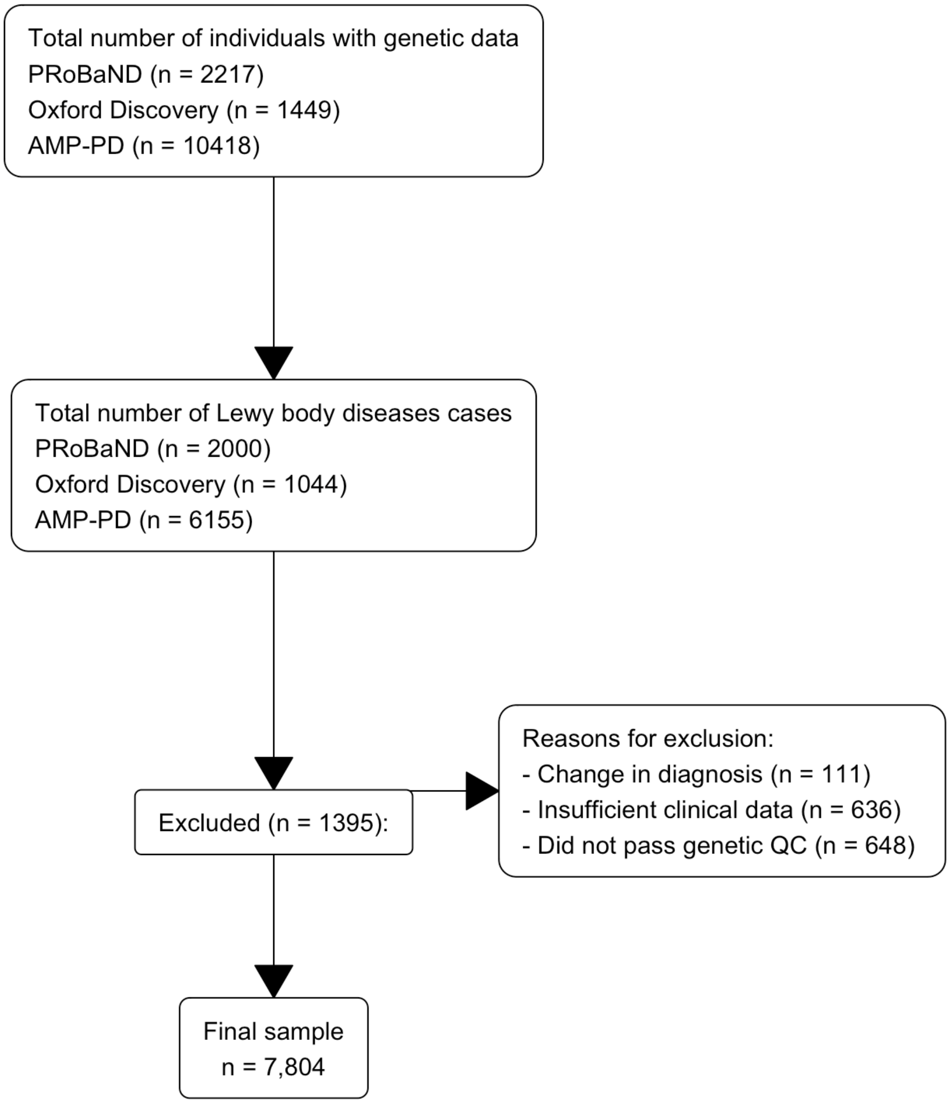

Supplementary Figure 1. Sample selection workflow

Samples were selected based on their diagnosis, and whether they passed clinical and genetic data quality control.

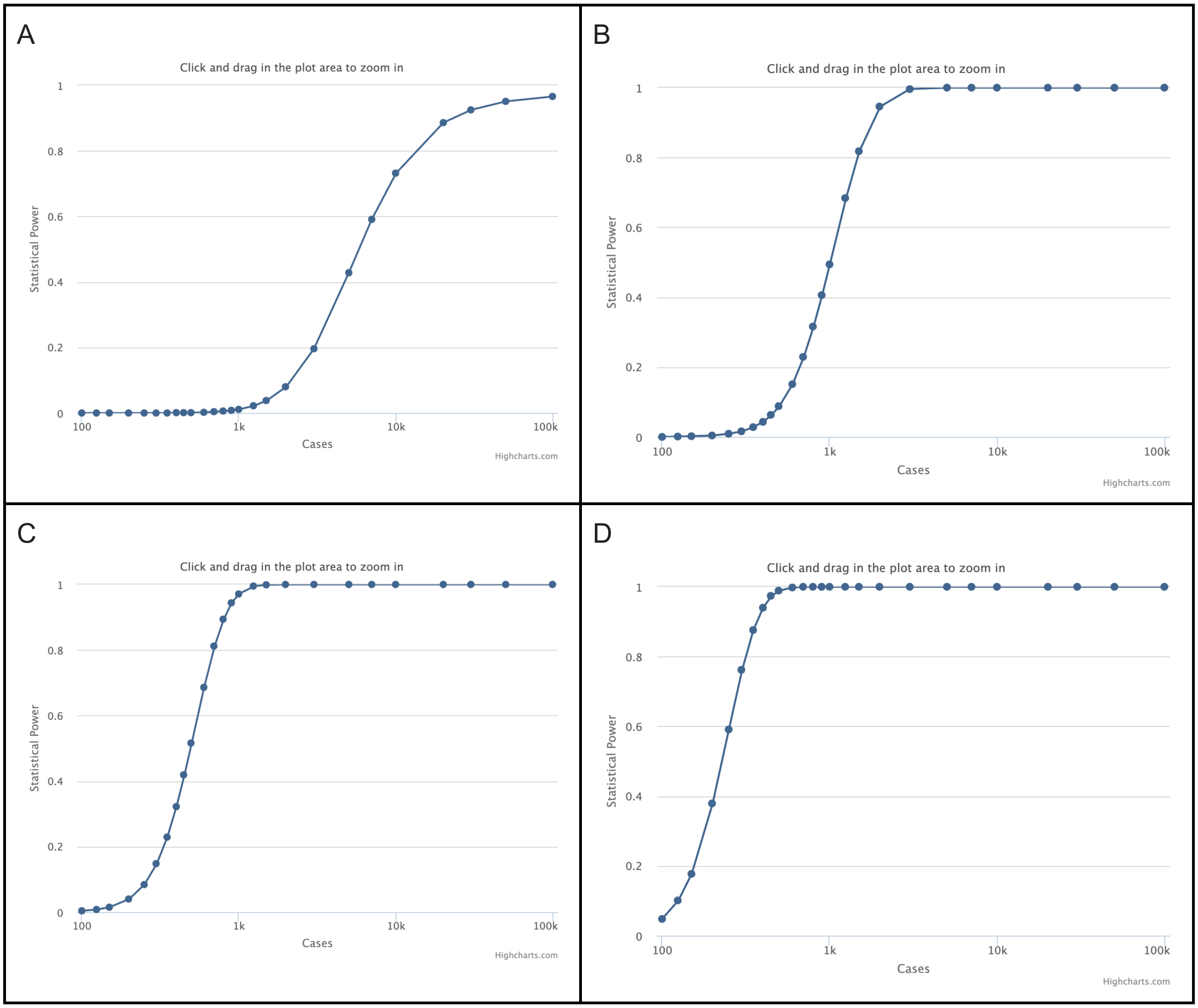

Supplementary Figure 2. Power calculation

Power calculation plots for detecting genetic variants associated with dementia, assuming an odds ratio of 1.2 (A), 1.4 (B), 1.6 (C), 1.8 (D) and a minor allele frequency of 0.15 under an additive model.

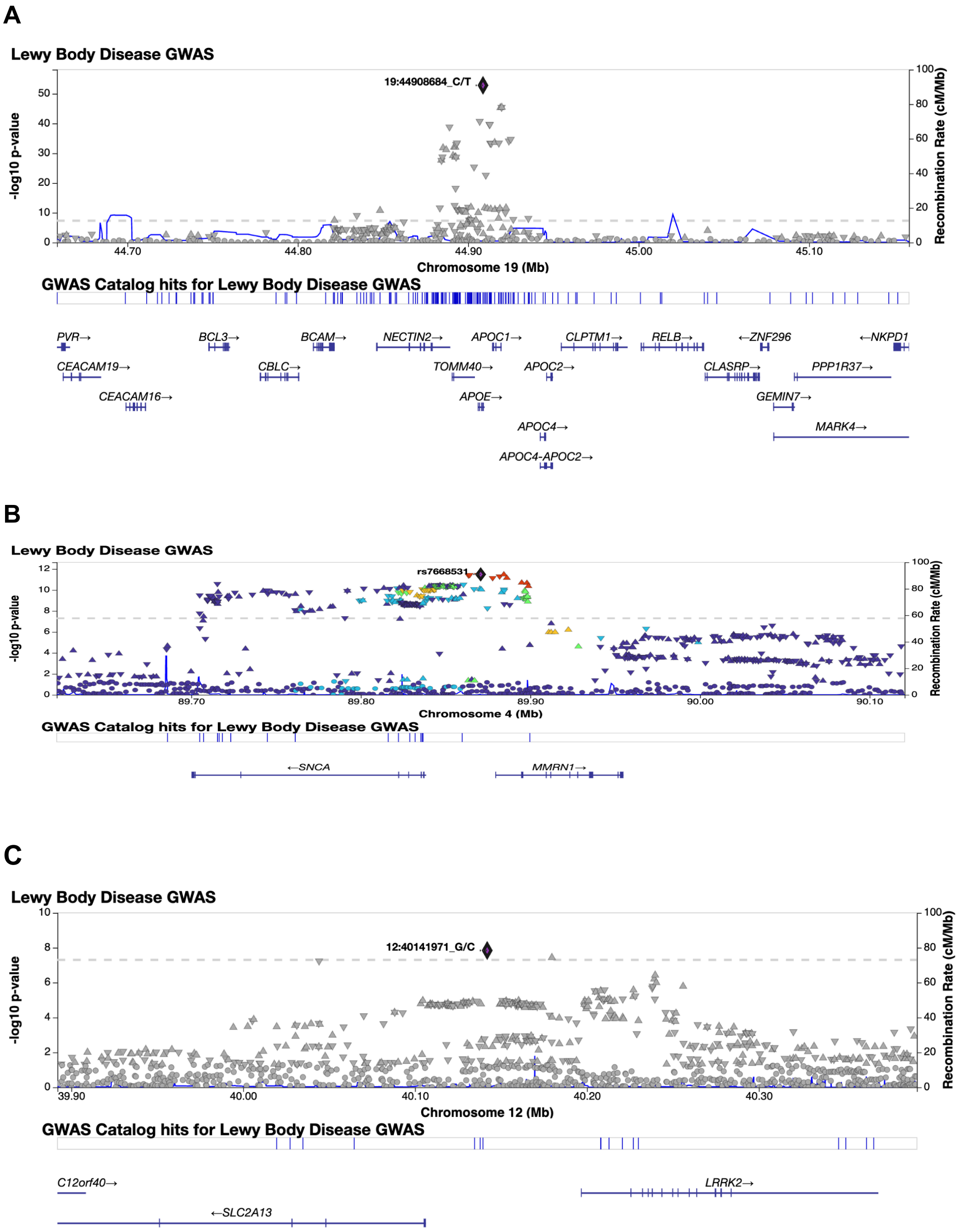

Supplementary Figure 3: Regional association plots of genome-wide significant SNPs

Regional association plots based on their genomic position for A) 19:44908684 (rs429358), B) 4:89870668 (rs7668531) and C) 12:40141971 (rs17442721). The index

variants are denoted by a purple diamond. Variants in the vicinity are color-coded to indicate the strength of linkage disequilibrium with the index variant (dark blue: $0 \le r2 < 0.2$; light blue: $0.2 \le r2 < 0.4$; green: $0.4 \le r2 < 0.6$; yellow: $0.6 \le r2 < 0.8$; red: $0.8 \le r2 \le 1$; grey: no r2 value available). Plots were generated in http://locuszoom.org/. SNP position and recombination rates are based on GRCh38.
